# The Potential Impact of Salivary IL-1 on the Diagnosis of Periodontal Disease: A Pilot Study

**DOI:** 10.3390/healthcare9060729

**Published:** 2021-06-13

**Authors:** Ji-Youn Kim, Ki-Rim Kim, Han-Na Kim

**Affiliations:** 1 Department of Dental Hygiene, College of Health Science, Gachon University, Incheon 21936, Korea; hoho6434@gachon.ac.kr; 2 Department of Dental Hygiene, Kyungpook National University, Sangju 37224, Korea; 3 Department of Dental Hygiene, College of Health and Medical Sciences, Cheongju University, Cheongju 28503, Korea

**Keywords:** biomarker, cytokine, diagnosis, periodontal disease, saliva

## Abstract

The aim of this study was to identify inflammatory cytokines as salivary biomarkers for periodontal disease. The subjects were 33 Korean adults aged 23 to 71 years. Using a multiplexed bead immunoassay called Luminex, the levels of inflammatory cytokines related to periodontal disease were evaluated. Oral examination for periodontal disease and gingival bleeding was conducted. With these two independent variables, differences in inflammatory cytokines were analyzed by an independent t-test and age-adjusted ANCOVA. Among the subjects, 21 had periodontal disease and 12 were healthy subjects. The gingival bleeding status was classified into low and high levels. Among 13 inflammatory cytokines in saliva, IL-1α, IL-1β, IL-4, IL-8, CCL2/MCP-1, CCL3/MIP-1α, and TNF-α were found to be significant biomarkers within the standard curve. The quantity of IL-1β was increased in subjects with high levels of gingival bleeding. IL-1α levels were increased in subjects with periodontal disease. After adjusting for age, the significant biomarkers for gingival bleeding and periodontal disease were IL-1β and IL-1α, respectively. Using the receiver operating characteristic (ROC) curve, IL-1β was confirmed as a significant biomarker. The sensitivity and specificity of IL-1β for predicting periodontitis were 88.24% and 62.5%, respectively. Therefore, IL-1 was found to be a significant biomarker for periodontal disease, and it could be used in the diagnosis of periodontal disease using saliva.

## 1. Introduction

Many people around the world experience periodontal disease. The Centers for Disease Control and Prevention (CDC) recently reported the prevalence of periodontitis in the United States (U.S.) at 47.2% of the adults aged 30 years and older. The prevalence increases with age, and 70.1% of adults 65 years and older have periodontal disease [1]. Starting with gingivitis, which is inflammation of the gingival tissue, periodontitis can progress to an irreversible state, leading to the destruction of periodontal tissues including periodontal ligaments and alveolar bone [2]. Generally, chronic periodontitis progresses slowly without acute pain. It gradually deteriorates the tissues around the teeth. Periodontal disease, a chronic inflammatory disease, is known to be caused by Gram-negative anaerobes or a bacterial complex. These bacteria can produce various cytokines such as interleukin (IL) and tumor necrosis factor-alpha (TNF-α), thus increasing polymorphonucleocyte (PMN) infiltration and matrix metalloproteinase (MMP) produced by osteoclasts. As a result, it can destroy tissues around the teeth or the alveolar bone. It also produces reactive oxygen species and peroxides [3]. Thus, it is important to detect and treat periodontal disease early so that inflammation caused by periodontal disease does not progress chronically.

As part of the effort to develop prognostic disease predictors, the discovery technology of biomarkers is being discussed. Biomarkers are molecular information based on the patterns of single molecules or molecules derived from DNA, RNA, metabolites, proteins, and protein fragments. They are indicators of changes caused by genetic or epigenetic changes in living organisms [4]. They can be identified easily in saliva by molecular clues that reflect systemic diseases or conditions. In addition, a saliva sample can be easily obtained non-invasively, unlike blood sampling. Therefore, the diagnosis and analysis of various diseases using saliva samples have attracted attention [5]. Recently, a large number of studies have reported that these approaches could successfully be used in the diagnosis of various systemic conditions such as cancer, autoimmune diseases, renal diseases, and diabetes [6,7,8,9,10,11]. Many researchers had emphasized the role and importance of saliva as a tool for the diagnosis of systemic and oral diseases [12]. However, the use of saliva for the diagnosis of periodontal disease is insufficient compared to that for other diseases [13].

Among the biomarkers used for the progression and diagnosis of periodontal disease, cytokines related to the inflammation associated with periodontal disease have received the most attention. The cytokines that can be identified in saliva related to inflammation include IL-1-beta (β), IL-6, IL-8, and TNF-α [14]. These cytokines in saliva could be used as biomarkers to diagnose periodontal disease or other oral diseases [15]. The amount of IL-1, IL-8, MMP-8, MMP-9, and MMP-13 in saliva samples from patients with periodontal disease and healthy controls have been analyzed and compared [16,17,18], and one study compared hemoglobin levels in blood samples with biomarkers in saliva samples from patients with periodontal disease [19]. Although several studies have been conducted to confirm the diagnosis of periodontal disease and disease progression using saliva, the biomarkers used in each study result were different [20,21,22]. Thus, basic research for diagnosing periodontal disease using the saliva of subjects of different races and countries needs to be conducted. This will help validate the use of saliva in a large, diverse patient population for the diagnosis of periodontitis [23]. Therefore, the purpose of this study was to analyze the saliva samples of 33 Korean adults over 20 years of age to select major biomarkers related to the inflammation of periodontal disease. The associations of biomarkers with gingival bleeding and the prevalence of periodontal disease were also determined. The results of this study provide basic data for diagnosing periodontal disease using saliva.

## 2. Materials and Methods

### 2.1. Study Participants

Information on prevention programs for the management of periodontal disease in the community was posted on the bulletin boards of the university and community centers in the region. Those who voluntarily participated were selected as participants. The inclusion criteria were adults aged 20 years or older, interested in periodontal disease treatment, and who had not received scaling or periodontal treatment during the past six months. The exclusion criteria were subjects with the loss of more than one-third of the posterior teeth, taking antibiotics due to disease, or with orthodontic devices. This study was conducted after obtaining approval from the Institutional Bioethics Committee (IRB No. 1041104-201910-BR-036-01).

Changes in inflammatory cytokines in saliva were identified according to gingival bleeding or periodontal disease. The subjects were classified according to the level of periodontal disease or gingival bleeding status. Depending upon the periodontal disease, the subjects with periodontal pockets of 4 mm or more in more than 30% of all teeth, subjects with clinical attachment levels (CALs) of 3 mm or more, and subjects with bleeding on probing (BOP) of 30% or more were classified as having severe periodontal disease. CAL refers to the value estimated using the periodontal probe for the position of the structure supporting the tooth. This is a criterion for determining the stability of teeth and the loss of the alveolar bone. BOP was measured in the mesial, central, and distal parts of the buccal and lingual side of each tooth (six parts per tooth) within about 30 s after probing. All third molars were excluded. If there was bleeding in the tooth, it was scored as 1 point, while 0 points indicated no bleeding [24]. To confirm the gingival bleeding status, the subjects with BOP of less than 30% were classified into the gingival bleeding-low group. If BOP was higher than 30%, the subject was classified into the bleeding-high group.

### 2.2. Saliva Collection

For stimulated saliva collection, the subjects chewed paraffin wax (Pinnacle bite modeling wax, Dentsply Sirona, Auckland, New Zealand) to collect more than 2 mL of irritant-provoked saliva. The subjects were instructed in advance to visit the hospital on an empty stomach as much as possible, without eating food or brushing with toothpaste at least an hour before saliva collection [25]. Ten minutes before the saliva collection, each subject completed a consent form for participation in the study and brushed without toothpaste for at least one minute using a new toothbrush provided by the researcher to reduce the number of microorganisms in the mouth before collecting saliva. The collected saliva was centrifuged at 10,000× *g* for 5 min at 4 °C using a centrifuge (Micro Refrigerated Centrifuge, Micro 17R, Hanil science, Gyeonggi-do, Korea) [26]. The supernatant was collected and stored in a −70 °C freezer (Ultra-Low Temperature Freezer, WUF-500, Daihan Scientific, Wonju-Shi, Korea) until analysis.

### 2.3. Cytokine Array in Saliva Samples

For the selection of biomarkers among the profile of inflammatory factors, we conducted a preliminary study using saliva samples from three subjects, a periodontally healthy (without periodontal disease) person, one with periodontal disease, and one with high BOP. The supernatants of the saliva samples were applied to a Human Inflammation Antibody Membrane (40 Targets) (Human Inflammation Antibody-Membrane, Abcam, UK) for the simultaneous detection of 40 human inflammatory factors according to the manufacturer’s instructions. The 40 target antibodies were eotaxin, eotaxin-2, granulocyte colony-stimulating factor, granulocyte-macrophage colony-stimulating factor, intercellular adhesion molecule 1, interferon-gamma (IFN-γ), I-309, IL-1α, IL-1β, IL-2, IL-3, IL-4, IL-6, IL-6sR, IL-7, IL-8, IL-10, IL-11, IL-12p40, IL-12p70, IL-13, IL-15, IL-16, IL-17A, interferon-inducible protein 10 (IP-10), chemokine (C-C motif) ligand 2 (CCL2)/monocyte chemoattractant protein 1 (MCP-1), MCP-2, macrophage colony-stimulating factor, monokine induced by IFN-γ, CCL3/ macrophage inflammatory protein (MIP)-1α, MIP-1β, MIP-1δ, regulated on activation normal T cell-expressed and secreted (RANTES), transforming growth factor (TGF)-β1, TNF-α, TNF-β, soluble TNF receptor 1 (sTNF-RI), sTNF-RII, platelet-derived growth factor (PDGF)-BB, and metallopeptidase inhibitor (TIMP)-2. The membranes were incubated overnight at 4 °C, washed with phosphate-buffered saline (PBS), and further incubated with paired biotinylated detection antibodies and streptavidin-horseradish peroxidase. The cytokine spots were detected with Amersham chemiluminescence reagents (GE Healthcare, Little Chalfont, UK).

### 2.4. Multiplex Fluorescent Bead Immunoassay of Saliva Samples

The levels of 13 selected cytokines (IFN-γ, IL-α, IL-1β, IL-2, IL-4, IL-6, IL-8, IL-10, IL-12p70, -17A, CCL2/MCP-1, CCL3/MIP-1α, and TNF-α) were analyzed in 33 saliva samples by multiplexed bead immunoassay (Luminex, Austin, TX, USA) and MAGPIX (Luminex Performance Human XL Cytokine Discovery Magnetic Panel, R&D systems, Minneapolis, MN, USA) according to manufacturer’s instructions. The results were analyzed using Bio-Plex (Bio-Rad, Hercules, CA, USA).

### 2.5. Statistical Analysis

The data were analyzed using IBM SPSS Statistics version 26.0 (IBM Co., Armonk, NY, USA). Frequency analysis was performed for the general characteristics. An independent t-test was performed to confirm the significant biomarkers in saliva samples according to the prevalence of periodontal disease and gingival bleeding status. An analysis of covariance (ANCOVA), which was adjusted for age, was used to confirm the significant differences. The levels of IL-1α, IL-1β, and TNF-α for detecting periodontal disease were determined in terms of sensitivity, specificity, and the area under the receiver operating characteristics (ROC) curves. The optimal combination of the parameters for screening for periodontitis was determined by the maximum sensitivity and specificity. The significance level was at *p* < 0.05.

## 3. Results

### 3.1. General Characteristics

Of the 33 subjects, 14 were women. The age of the subjects ranged from 23 to 71 years, with a mean age of 42.43 years. Twenty-one were patients with periodontal disease and twelve were healthy adults. Twenty-five patients had a high level of gingival bleeding. Of all subjects, four had systemic diseases, including diabetes, hyperlipidemia, and cardiovascular disease (Table 1).

### 3.2. Screening of Periodontal Disease-Related Biomarkers in Saliva Samples

**Figure 1:**
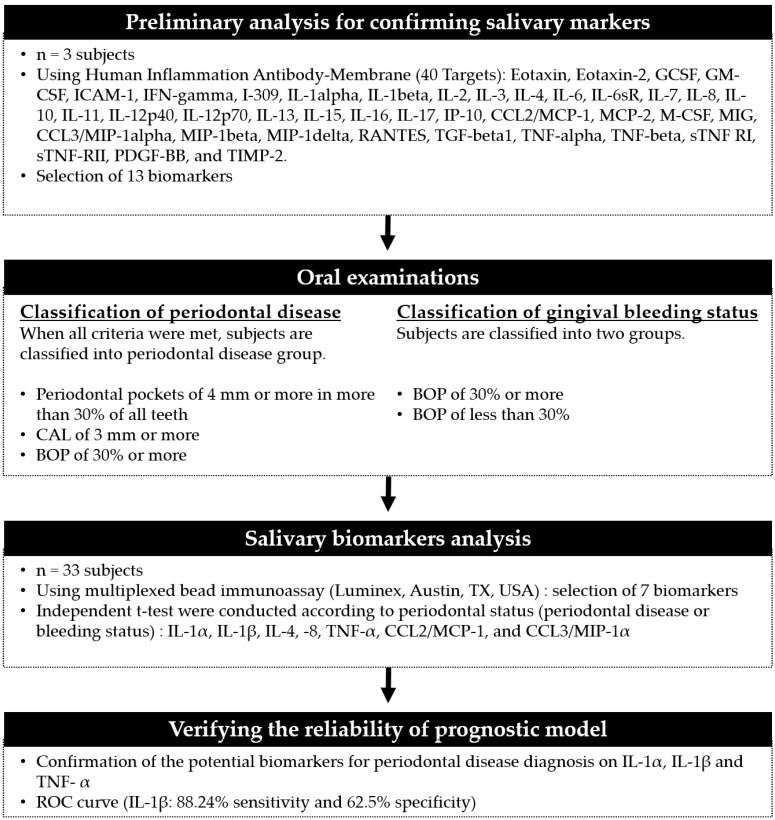
Flowchart showing the study design and applied saliva analysis.

We conducted a preliminary screening to select the periodontal disease-related biomarkers among the various inflammatory cytokines. Thirteen cytokines showing changes according to the prevalence of periodontal disease and gingival bleeding were selected among the 40 human inflammatory factors. In particular, the analysis revealed that the levels of IL-1α, IL-1β, IL-4, IL-8, IL-10, IL-12p70, IL-17, CCL2/MCP-1, CCL3/MIP-1α, and TNF-α in the saliva of patients with periodontal disease were more than twice as high as those in the saliva of subjects without periodontal disease (data not shown). Quantitative data were obtained from the Luminex assay. IFN-γ, IL-2, IL-6, IL-10, IL-12p70, and IL-17A were excluded because they extrapolated from the standard curve. Finally, seven salivary biomarkers, including IL-1α, IL-1β, IL-4, IL-8, CCL2/MCP-1, CCL3/MIP-1α, and TNF-α remained. We confirmed the correlation between each biomarker and the periodontal status (Figure 1).

### 3.3. Potential Salivary Biomarkers for Predicting Gingival Bleeding

We investigated whether there was a significant difference in each biomarker according to gingival bleeding status. IL-1β and TNF-α had a significant difference among seven cytokines (*p* = 0.025 and *p* = 0.028, respectively). In subjects with gingival bleeding of 30% or more, the mean IL-1β level was 265.31 pg/mL, which was higher than 81.80 pg/mL in the subjects with less gingival bleeding. The TNF-α levels were lower in the high gingival bleeding status group. The levels of IL-1α, IL-8, and IL-4 were higher in the gingival bleeding-high group than those in the gingival bleeding-low group. There was no significant difference between the two groups. CCL2 showed little difference according to gingival bleeding status. The CCL3 levels were relatively high in healthy subjects with less gingival bleeding. After age adjustment, IL-1β was a significant biomarker according to gingival bleeding status (*p* = 0.049) (Table 2).

### 3.4. Potential Salivary Biomarkers for Predicting Periodontal Disease

We investigated whether there was a significant difference between each biomarker and the prevalence of periodontal disease. Among seven cytokines, statistically significant differences in IL-1α and TNF-α levels were detected (*p* = 0.048 and *p* = 0.045, respectively). In subjects with periodontal disease, IL-1α was found to be 590.78 pg/mL, which was higher than 343.60 pg/mL found in healthy subjects. The level of TNF-α was confirmed to be 13.64 pg/mL, which was lower than 15.25 pg/mL in healthy subjects. The levels of IL-1β, IL-8, and IL-4 were higher in the patients with periodontal disease than in the healthy subjects, although the differences were not statistically significant. The levels of CCL2/MCP-1 and CCL3/MIP-1α were high in the healthy subjects. After adjusting for age, only IL-1α was significantly higher in the periodontal disease subjects (*p* = 0.037) (Table 3).

### 3.5. ROC Curves of IL-1α, IL-1β, and TNF-α for Periodontal Disease Diagnosis

**Figure 2:**
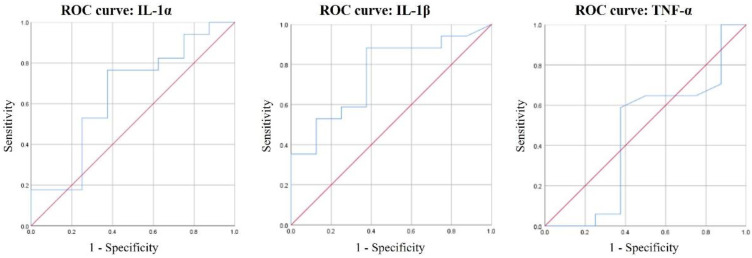
ROC curves of IL-1α, IL-1β, and TNF-α for periodontal disease diagnosis.

IL-1α, IL-1β, and TNF-α as predictive biomarkers for the diagnosis of periodontal disease were analyzed by ROC curves (Figure 2). The only significant biomarker was IL-1β (*p* = 0.044). The threshold value for IL-1β showing 88.24% sensitivity and 62.5% specificity was 69.29 pg/mL.

## 4. Discussion

In periodontal disease, the patient cannot check the severity of the disease as it progresses. Continuous treatment is required to regenerate the tissue around a tooth that has been lost. Therefore, it is very important to diagnose periodontal disease in its early stage. Unlike general systemic diseases, the development of a kit for the diagnosis of oral diseases has not been accomplished. The development of over-the-counter drug-type periodontal disease diagnostic kits that can be easily accessed by the public can increase visits to medical institutions for early detection and treatment.

In this study, we used human inflammation antibody arrays to identify the profile of inflammatory factors related to periodontal disease in the saliva of three subjects. Among 40 human inflammatory factors screened, 13 were detected. We conducted a bead-based multiplex immunoassay to measure the levels of 13 selected factors in 33 saliva samples. Until now, enzyme-linked immunosorbent assays (ELISAs) have been used in many studies to analyze the inflammatory enzymes expressed by cells or bacteria. ELISA has the disadvantage that only one substance can be identified at a time. However, Luminex used in this study can analyze various substances at once, even when there are many samples or biomarkers to be analyzed. Therefore, it has the advantage of enabling multiple analyses of a small amount of sample and reducing the cost and time required [27,28]. In addition, recent studies on the clinical application of biomarkers have demonstrated that Luminex multiplex assays were a useful tool to validate and quantitate protein levels in saliva [29,30]. According to a study comparing the validation of Luminex and ELISA kits, Pearson’s correlation coefficient (r) between TNF-α and IL-1β were 0.937 and 0.838, respectively, confirming a high correlation [31]. However, research using saliva for Luminex analysis is lacking, so there is a limit to directly comparing the quantitative research results.

Among the selected 13 factors, IL-1α, IL-1β, IL-4, IL-8, CCL2/MCP-1, CCL3/MIP-1α, and TNF-α were found to have significant values within the standard curve. IL-1 plays an important role in inflammation and the immune response. It induces tissue inflammation and bone resorption. It is produced and released locally within periodontal lesions. Thus, it can be an index of the destruction of periodontal tissue [32,33]. IL-4 is produced by CD4^+^ T lymphocytes induced by an immune response [34]. Compared to other cytokines, its direct association with periodontitis has not been confirmed. However, it is found in the serum of patients with periodontitis. In the present study, IL-4 was found in the saliva. However, its association with periodontal disease could not be confirmed, which is consistent with the results of previous studies. IL-8 is a cytokine that can induce inflammation. It can also induce adhesion molecule expression, chemotaxis, and neutrophil activation and cause bone resorption [35]. Previous studies have reported that IL-8 levels were correlated with periodontitis because IL-8 levels in the gingival crevicular fluid of periodontitis patients and periodontal tissues were increased. The importance of IL-8 in the pathogenesis of periodontitis has been suggested [36]. Thus, further research on IL-8, which was identified in saliva, is necessary. CCL2/MCP-1 and CCL3/MIP-1α are substances primarily identified in patients with rheumatoid arthritis [37]. When vascular endothelial cells are damaged, monocytes pass through endothelial cells and move into blood vessels. Monocytes located in blood vessels have characteristics of macrophages. Monocytes secrete various inflammatory cytokines, tissue factors, growth factors, and MMPs. The cytokines and growth factors released from macrophages can induce various types of immune responses including T cells [38]. TNF is known to be produced when human gingival fibroblasts are stimulated. It can cause bone resorption and the destruction of periodontal tissue [39,40]. Many studies have reported the association of cytokines with periodontal disease, supporting the results of the present study, showing that these substances were present in saliva samples.

The level of each salivary biomarker was identified according to the gingival bleeding status. Compared to the low gingival bleeding group, the levels of IL-1β in the high gingival bleeding group were significantly higher, and the levels of TNF-α were significantly lower in the high gingival bleeding group. However, the ANCOVA results, which were adjusted for age, showed that IL-1β was biomarker related to gingival bleeding status. There is a lack of studies on the salivary biomarkers associated with gingival bleeding. Regarding gingival and dental plaque indices, Lee et al. [41] reported that high levels of IL-6 and MMP-1 had the ability to predict high gingival inflammation. In addition, Syndergaard et al. [42] presented that MIP-1α and PGE2 concentrations in saliva were significantly higher in the gingivitis group compared to healthy group; however, IL-1β, IL-6, and MMP-8 concentrations were unable to distinguish gingivitis from health. They focused on gingivitis, a reversible condition characterized by elevated BOP, plaque index, and gingival index scores. Therefore, the present study is not directly comparable with previous studies because it may include some subject with alveolar bone loss or advanced periodontal disease in the gingival bleeding high group.

The level of each salivary biomarker was identified according to the prevalence of periodontal disease. A significant association between IL-1α and TNF-α and the prevalence of periodontal disease was confirmed. Many studies have indicated that IL-1 is a cytokine associated with periodontal disease. In a systematic review of saliva cytokine changes before and after non-surgical periodontal therapy, IL-1β, TNF-α, MMP-8, and MMP-9 were found to be the most important cytokines [43]. Romero-Castro et al. [44] confirmed that TNF-α in the gingival crevicular fluid was significantly higher in the bone loss group. High levels of IL-1, TNF-α, and IL-6 have been also found in the gingival crevicular fluid and plasma of patients with periodontal disease. In addition, the relationship between gene polymorphisms of IL-1α and IL-1β and periodontal disease were significantly different according to the severity of periodontitis [45]. Thus, the accumulated evidence from these studies have suggested IL-1 and TNF-α as important biomarkers. However, in the present study, the ANCOVA results, which were adjusted for age, showed that only IL-1α was biomarker related to periodontal disease. The level of TNF-α was not significantly changed according to periodontitis status. This might be due to the following reasons. There might have been a difference in the degree of periodontal disease progression among the subjects included in this study and previous studies. There might also have been different systemic diseases in the subjects included in the previous studies. Singh et al. [46] suggested that TNF-α in saliva was a potential marker of periodontal tissue destruction. They compared the following groups: periodontal disease patients with type 2 diabetes, periodontal disease patients who smoked 10 or more cigarettes per day, periodontal disease patients with a periodontal pocket depth of 5 mm or more and a CAL of 2 mm, and healthy subjects. They found that the average TNF-α saliva values were in the order of periodontal disease patients with diabetes > periodontal disease patients who smoked > periodontal disease patient > healthy subjects. Varghese et al. [47] reported that the average level of TNF-α was higher in patients with chronic periodontitis than in healthy controls. However, the levels were not significantly different between patients with acute periodontitis and healthy controls. Thus, the association between TNF-α in saliva and periodontal disease might depend upon the health status of the study subjects and the severity and progression of periodontal disease.

The ROC curve showed that among the predictive factors of IL-1α, IL-1β, and TNF-α, the only significant biomarker was IL-1β (*p* = 0.044). We could not confirm a significant association of IL-1α and TNF-α with periodontal disease in the present study. The usefulness of IL-1β as a biomarker of periodontal disease was confirmed through sensitivity and specificity analysis. In this study, the threshold value for IL-1β (69.29 pg/mL) was relatively low compared to mean value of healthy subjects. This might be because the healthy subjects were mostly non-smokers and the average IL-1β value was low in the younger age group. Sanchez et al. [18] reported that with a selected threshold of 212 pg/mL, salivary IL-1β predicted periodontitis with 78% sensitivity and 100% specificity. It is difficult to determine the threshold for diagnosis using the ROC curve with a single value. Therefore, it is challenging to suggest an absolute value of IL-1β for predicting periodontitis. The value of making a clinical diagnosis using an ROC curve varies depending upon the measurement environment and the target. However, if the results of each biomarker are accumulated, it is believed that basic data for a diagnostic kit for screening periodontitis can be presented.

In this study, the saliva samples of 33 subjects were analyzed to identify the biomarkers related to inflammation and periodontal disease. However, it was not possible to analyze many samples because the number of subjects who gave consent for participation was limited. In addition, since the subjects participating in the study had a large age range, it was impossible to subdivide the subjects according to age and perform analysis due to the insufficient number of subjects. Additionally, among them, subjects with systemic diseases such as diabetes, cardiovascular disease, and hyperlipidemia were included. These diseases might have affected the results of the study. However, efforts were made to confirm inflammatory cytokines associated with periodontal disease using saliva samples as much as possible in the present study. Further studies should include many subjects and perform subgroup analysis according to age and systemic disease. In addition, a recent study [48] reported that a combination of multiple biomarkers rather than the use of single ones and the ratios of key biomarkers was important. A recent systematic review reported that the combination of MIP-1α, IL-1β, IL-6, and MMP-8 was acceptable as biomarkers for diagnosing periodontal disease [49]. It implies that vigorous research on a single biomarker or a combination of universally reliable biomarkers is required for definitive early diagnosis.

The present study attempted to identify the major biomarkers by comparing two important variables, gingival bleeding and the prevalence of periodontal disease. The results will help strengthen the foundation of salivary biomarker research for the early detection of periodontal disease.

## 5. Conclusions

This study analyzed the saliva samples of Korean adults to identify periodontal disease-related inflammatory cytokines. From the results adjusting for age, the significant biomarkers were IL-1β and IL-1α for gingival bleeding and periodontal disease, respectively. This, together with the high sensitivity and specificity of IL-1β for periodontal disease reported here, suggests that IL-1 could be useful surrogate indicators for the presence of periodontal disease and inflammation. Thus, they could be used in the diagnosis of periodontal disease using saliva.

## Figures and Tables

**Table 1:** Characteristics of the subjects.

Variables	Items	N
**Gender**	Male Female	19 14
**Age (year)**	≤39 40–49 50–59 ≥60	6 7 14 6
**Periodontal disease**	Yes No	21 12
**Gingival bleeding status**	High Low	25 8
**Systemic disease**	Yes No	4 29
**Total**		33

**Table 2:** Salivary cytokines associated with gingival bleeding.

Biomarkers	Gingival Bleeding Status	Mean (pg/mL)	SD	*p*-Value *	*p*-Value **
IL-1α	High	729.22	497.16	0.170	0.333
	Low	438.89	254.64		
IL-1β	High	265.31	199.52	0.025	0.049
	Low	81.80	46.60		
IL-8	High	444.94	273.73	0.442	0.752
	Low	349.88	187.71		
TNF-α	High	13.46	3.80	0.028	0.374
	Low	15.40	6.76		
IL-4	High	3.46	1.17	0.683	0.826
	Low	3.19	1.31		
CCL2/MCP-1	High	268.60	152.37	0.992	0.593
	Low	269.35	136.38		
CCL3/MIP-1α	High	25.50	9.92	0.556	0.957
	Low	29.12	12.83		

* independent *t*-test. ** ANCOVA, adjusted for age.

**Table 3:** Salivary cytokines associated with periodontal disease.

Biomarkers	Periodontal Disease	Mean (pg/mL)	SD	*p*-Value *	*p*-Value **
IL-1α	Y	590.78	344.72	0.048	0.037
	N	343.60	188.30		
IL-1β	Y	216.98	180.81	0.106	0.095
	N	94.55	96.93		
IL-8	Y	459.89	389.10	0.881	0.622
	N	435.10	303.45		
TNF-α	Y	13.64	4.07	0.045	0.837
	N	15.25	6.82		
IL-4	Y	3.54	1.30	0.547	0.777
	N	3.16	1.61		
CCL2/MCP-1	Y	315.12	203.11	0.335	0.460
	N	407.98	264.82		
CCL3/MIP-1	Y	30.52	10.23	0.402	0.302
	N	34.56	11.74		

* independent *t*-test. ** ANCOVA, adjusted for age.

## Data Availability

The data that support the findings of this study are available from the National Research Foundation (NRF) funded by the Ministry of Science and ICT (MSIT), but restrictions apply to the availability of these data, which were used under license for the current study, and so are not publicly available.
